# Prevention is better: the case of the underutilized failure mode effect analysis in patient safety

**DOI:** 10.1186/s13584-016-0131-2

**Published:** 2017-02-20

**Authors:** Lewis Goodrum, Prathibha Varkey

**Affiliations:** 1 Yale New Haven Health, 2 Howe St., New Haven, CT 06511 USA; 2 Yale New Haven Health Northeast Medical Group, 99 Hawley lane, 3rd Flr, Stratford, CT 06614 USA

**Keywords:** Failure mode and effects analysis, Patient safety, Prospective hazard analysis, Healthcare management, Healthcare failure mode and effects analysis, Clinical quality, Patient safety tools

## Abstract

Prospective hazard analysis methodologies, like failure modes and effects analysis (FMEA), have been tried and tested in the engineering industry and are more recently gaining momentum in healthcare. Considering FMEA’s evidence based successes, this commentary makes the case that healthcare is underutilizing the methodology by relying on retrospective hazard analysis. Healthcare leaders should determine where prospective hazard analysis principles could be better built into care delivery planning and processes that will enhance patient safety.

## Commentary

The field of medicine has traditionally been built on a reactive approach of healing illness, rather than preventing it. Likewise, healthcare improvement has traditionally focused on improving existing processes and analyzing errors after they have occurred. In contrast, to arrive at the advanced safety levels, as seen in high reliability organizations, there is a need for commitment of top management commitment for the time and resources needed to advance the safety culture, and for trust in front-line employees to provide key input about the safety changes needed [1].

In a recent IJHPR article, Fanny Ofek et al. report on the use of prospective hazard analysis to implement policy change for intravenous potassium chloride (IV KCl) administration. They review the impetus for change, criteria for selection of the failure mode and effect analysis (FMEA) methodology, and the patient safety enhancements that resulted from successful utilization of the methodology [2]. The authors discussed the motives for changing the policy and administration methods for IV KCl, including to reduce the risk of accidental overdose of high-alert medications. The matter was urgent, impacted highly on patient safety, and was a notable change in treatment patterns. Utilization of the FMEA methodology was selected because Ofek et al. decided to prospectively intervene in harm reduction before the new policy was implemented.

Organizations always face competing priorities. Matrix and prioritization grids can provide assistance when trying to select patient safety projects in that context. They involve assessing criteria, such as urgency, level of impact, likeliness of occurrence, complexity, and anticipated value of the safety project [3–5]. FMEA methodology is similar to the matrix and prioritization grids in that it provides a systematic prioritization of factors. It differs uniquely, however, from other patient safety methodologies by being prospective, rather than retrospective.

A commonly used tool in the field of engineering, the FMEA has been used since the early 1990s to increase safety and consumer satisfaction, reduce cost, and reduce product time to market [6]. Several institutions, such as the Mayo Clinic, have adopted engineering principles more rapidly by including their engineering department as a part of the care team to bring process expertise to the table with clinicians. The engineers facilitate prospective hazard analysis when implanting new procedural techniques or adopting new technologies. In general, the healthcare industry has lagged behind other industries in adopting prospective hazard analysis by frequently relying on retrospective event analysis.

Since the early 2000s, the Veteran’s Administration National Centre for Patient Safety has utilized a health-care specific version of FMEA called healthcare failure mode and effect analysis (HFMEA) [7]. While FMEA and HFMEA work to accomplish the same goal of proactive hazard analysis, HFMEA modifies the FMEA hazard scoring processes and decision-making steps to bring more specificity to healthcare settings.

Retrospective event analysis, including root cause analysis, is similar to FMEA in that steps of process mapping, data analysis, and cause analysis must occur. One important distinction of root cause analysis from FMEA is that harm has already occurred and it is reactionary. In being reactive and retrospective, we continue to rely on analyses of errors after they have occurred, while not investing in building new safety processes and devices. In order to accelerate achievement of the patient safety ideal of zero preventable adverse events, prospective measures can and should be used with greater frequency.

Challenges involved in implementing FMEA and other prospective hazard analyses include investment in time and resources, training, and management commitment [6, 8]. Considering the FMEA process step of assembling a multidisciplinary action team, including patient-facing caregivers, a modest time commitment as well is necessary to map the process and identify failure modes.

## Conclusions

Ofek et al., through successful risk avoidance using the FMEA methodology, achieved much more than clinical quality and safety enhancements; they also increased the functional health and well-being of the population they served, reduced costs associated with complications, and achieved clinician and patient satisfaction with the process. Their outcomes demonstrate the value proposition behind preventing errors using prospective hazard analysis.

With recent publications suggesting that the epidemic of medical errors (now ranked third in leading causes of death in the US) persists, a re-look and overhaul of our current patient safety tools and processes seems imperative [9]. Future research should focus on determining the areas in healthcare where FMEA principles could be built into processes and devices prior to implementation, while continuing retrospective root cause analyses in situations where errors have occurred despite precautions.

## Abbreviations

FMEA: Failure mode and effect analysis
HFMEA: Healthcare failure mode and effect analysis
IV KCl: Intravenous potassium chloride
